# Water treadmill training attenuates blood-spinal cord barrier disruption in rats by promoting angiogenesis and inhibiting matrix metalloproteinase-2/9 expression following spinal cord injury

**DOI:** 10.1186/s12987-020-00232-1

**Published:** 2020-11-25

**Authors:** Xinwang Ying, Qingfeng Xie, Shengcun Li, Xiaolan Yu, Kecheng Zhou, Jingjing Yue, Xiaolong Chen, Wenzhan Tu, Guanhu Yang, Songhe Jiang

**Affiliations:** 1 Department of Physical Medicine and Rehabilitation, The Second Affiliated Hospital and Yuying Children’s Hospital of Wenzhou Medical University, Zhejiang, Wenzhou, 325000 China; 2 Department of Intelligent Rehabilitation International (Cross-Strait), Alliance of Wenzhou Medical University, Zhejiang, Wenzhou, 325000 China

**Keywords:** Spinal cord injury, Blood-spinal cord barrier, Water treadmill training, Vascular regeneration, Matrix metalloproteinase-2/9

## Abstract

**Background:**

The permeability of the blood-spinal cord barrier (BSCB) is mainly determined by junction complexes between adjacent endothelial cells (ECs), including tight junctions (TJs) and adherens junctions (AJs), which can be severely damaged after spinal cord injury (SCI). Exercise training is a recognized method for the treatment of SCI. The destruction of the BSCB mediated by matrix metalloproteinases (MMPs) leads to inflammation, neurotoxin production, and neuronal apoptosis. The failure of new blood vessels to effectively regenerate is also an important cause of delayed recovery after SCI. For the first time, we introduced water treadmill training (TT) to help SCI rats successfully exercise and measured the effects of TT in promoting recovery after SCI and the possible mechanisms involved.

**Methods:**

Sprague-Dawley (200–250 g) rats were randomly divided into the following three groups: sham operated, SCI, and SCI + TT. Animals were sacrificed at 7 or 14 days post-surgery. The degree of neurological deficit, tissue morphology and BSCB permeability were assessed by the Basso-Beattie-Bresnahan (BBB) motor function scale and appropriate staining protocols, and apoptosis, protein expression and vascular EC ultrastructure were assessed by TUNEL staining, Western blotting, immunofluorescence and transmission electron microscopy (TEM).

**Results:**

Our experiments showed that TT reduced permeability of the BSCB and decreased structural tissue damage. TT significantly improved functional recovery when compared with that in the SCI group; TJ and AJ proteins expression increased significantly after TT, and training reduced apoptosis induced by SCI. TT could promote angiogenesis, and MMP-2 and MMP-9 expression was significantly inhibited by TT.

**Conclusions:**

The results of this study indicate that TT promotes functional recovery for the following reasons: TT (1) protects residual BSCB structure from further damage, (2) promotes vascular regeneration, and (3) inhibits MMP-2/9 expression to mitigate BSCB damage.

## Background

Spinal cord injury (SCI) is responsible for heavy societal and economic burdens [1]. Currently, SCI can only be relieved by surgery, and cannot be cured [2]. Endothelial cells (ECs), the basement membrane, pericytes and the terminal foot processes of astrocytes constitute the blood-spinal cord barrier (BSCB), which normally protects the parenchyma [3, 4]. After SCI, barrier integrity is compromised by the disruption of interendothelial tight junctions (TJs) and adherens junctions (AJs) as well as overall mechanical damage to vessels. This compromise in the BSCB results in the infiltration of immune cells and neurotoxic products, causing the death of nerve cells, and permanent neurological dysfunction [5–7]. Therefore, there is a crucial need to identify interventions that can effectively prevent BSCB destruction after SCI.

Matrix metalloproteinases (MMPs), a family of zinc-containing peptidases that degrade and reshape the extracellular matrix and other extracellular proteins, play a key role in barrier function [7, 8]. Studies have shown that MMPs exacerbate the destruction of the BBB/BSCB under pathological conditions, including SCI [8, 9]. Two important members of the MMP superfamily are MMP-2 and -9 [10]. MMP-9 can induce BSCB-related protein degradation, and the upregulation of MMP-2 can lead to initial opening of the BBB/BSCB [11, 12]; in contrast, blocking MMP-9 activity can protect against vascular permeability [9]. MMP-2/9 expression has been detected up to 7 days after SCI [13]. Vascular endothelial growth factor (VEGF) is a highly specific vascular EC growth factor that promotes vascularisation, proliferation, and angiogenesis [14]. Additionally, VEGF is a necessary angiogenic factor for embryonic development and neovascularization under many pathological conditions [15]. Several molecules have been demonstrated to be angiogenic in vivo, but only VEGF is considered to be a secretory mitogen specific to vascular ECs. At present, VEGF is the most likely target for the study of vascular growth dynamics [16, 17].

Previous studies on this topic have mainly focused on drug treatment after SCI [18, 19]. However, these treatments often have specific side effects, affecting patient quality of life. Therefore, it is important to identify a safe, effective and healthy treatment for patients with SCI. Exercise training is a non-traumatic rehabilitation method that can promote the functional recovery of paralyzed muscles [20–23]. To date, most studies have focused on the effects of drug therapy on the neurovascular system after SCI [24, 25], but ignored the role of exercise training in protection and functional recovery of the vascular system. Based on this fact and starting from clinical practice to simulate exercise rehabilitation in patients, our experimental team has designed and invented the first water treadmill that is suitable for rats to exercise after SCI. Compared with swimming exercise, water treadmill training(TT) causes rats to passively and forcibly exercise at the initial stage, after which rats actively follow the treadmill in the middle and later stages of training. It is difficult for rats to perform rehabilitation exercise on an ordinary treadmill due to its high resistance. Our water treadmill equipment combines swimming and TT, which addresses the above shortcomings. The protective effects of TT on SCI have not been reported in the literature, and its effects on the BSCB are unclear. The aim of these experiments was to measure the protective effects of TT on the BSCB after SCI and investigate the mechanisms involved.

## Method

### Antibodies

Anti-MMP-9, anti-MMP-2, and anti-Tubulin antibodies were purchased from Proteintech (Rosemont, IL, USA). Anti-VEGF, anti-BrdU, anti-Laminin and anti-β-Actin antibodies were purchased from Abcam (330 Cambridge Science Park, Cambridge, UK). Anti-p120-Catenin, anti-β-Catenin, anti-ZO-1, anti-Occludin, anti-Claudin-5 and anti-CD31 antibodies were purchased from Affinity (OH, USA). An in situ cell death detection kit was purchased from Roche Molecular Biochemicals.

### Animals

A total of 123 adult male Sprague-Dawley rats (200–250 g) were purchased from the Shanghai Laboratory Animal Center. The animal-related protocols had been approved by the Animal Research Committee of Wenzhou Medical University. All animals were housed in a controlled environment and regularly fed food and water. Rats were randomly divided into the following three groups: sham-operated (n = 41; group S), SCI (n = 41; group M), and SCI + TT (n = 41; group TM).

### SCI modelling

Rats were anaesthetized with 2% pentobarbital sodium (30 mg/kg) and shaved, after which a 2-cm incision was made to expose the T10 segment of the spinal cord [26]. The exposed site was impacted with a New York University (NYU) Impactor (10 g × 20 cm) in all groups except group S. Lower limb trembling contractions and tail wagging showed that SCI modeling had been successful. Finally, the wound was sutured and disinfected with iodophor, and the rats were allowed to recover from the anaesthetic. Over the following days, the bladder was emptied manually every morning and evening.

### Water TT

Our research group provided the initial design for the water treadmill (Wenzhou Xinglong Stainless Steel Co., Ltd, Zhejiang, China) and submitted it for patenting. The rats were given adaptive training for three days before SCI. The surface of the water was adjusted to the xiphoid process of the rat sternum, the water temperature was set at 30 °C, and the speed of the water treadmill was maintained at 10–15 m per minute. Training started one day after SCI. Rats in the training group(TM) began training that lasted for 7 or 14 days (5 min/round, 3 rounds in total, 5-min interval between rounds) (An attached video file shows the movement of the rats on the water treadmill [see Additional file 1]).

### Behavioural tests

Two independent examiners who were blinded to the treatment groups conducted the Basso-Beattie-Bresnahan (BBB) motor functional scale test in an open field. The maximum total BBB score was 21 points, and the higher the score was, the closer the animal’s motor function was to normal [27].

## Evaluation of BSCB permeability

### Water content

At 7 or 14 d after SCI, 2% sodium pentobarbital was intraperitoneally injected to anaesthetize the animals (*n* = 5), after which the rats underwent cardiac perfusion with 0.9% normal saline and 0.5 cm of the T10 spinal cord segment was removed. The degree of oedema in this segment was assessed by the dry and wet weight method as previously reported [28, 29].

### Evans blue (EB) dye assay

According to previously reported methods [4, 8], rats (*n* = 5) were injected with EB dye (4 ml/kg) by the tail vein at 7 or 14 d after SCI to assess BSCB permeability, followed by treatment with 2% sodium pentobarbital anaesthesia 2 h later and 0.9% saline perfusion. Tissues containing T10 were soaked in N,N′-dimethylformamide at 50 °C for 72 h. The concentration of EB dye in the samples was determined based on a standard curve (μg/g). Tissues were cut into 15-μm thick sections with a freezing microtome at − 20 °C, and then analysed. Quantitative data analysis was performed with ImageJ software.

### Haematoxylin–eosin (HE) staining

Briefly, T9–T11 spinal cord tissue was removed from the rats at 7 or 14 d after SCI, and stored in 4% paraformaldehyde for 24 h (4 °C). The spinal cord tissue was immersed in a 0.1 M phosphate buffer solution and 30% sucrose solution overnight (4 °C). Successive sections (15-μm thick) were frozen and stored for subsequent HE staining.

### Western blot analysis

Tissues containing T10 segments were put into a collection tube containing a mixture of phenylmethanesulfonyl fluoride (PMSF) and RIPA lysis buffer (100:1) and then microcentrifuged at 12,000 rpm for 5 min at 4 °C. We extracted the supernatant and calculated the protein concentration with a BCA protein assay kit. The mixed solution was heated to 100 °C for 10 min. After electrophoretic transfer to membranes, they were incubated with the appropriate primary (anti-p120-Catenin, anti-β-Catenin, anti-ZO-1, anti-Occludin, anti-Claudin-5, anti-MMP-9, anti-MMP-2, anti-VEGF, anti-Tubulin, or anti-β-Actin) and secondary antibodies, and the signal was digitally quantified.

### Immunofluorescence staining

After the sections had been dried, they were washed 3 times for 15 min. They were treated with non-immune goat serum for 1 h and then incubated with the following primary antibodies for 50 min at room temperature: rabbit anti-Occludin antibody (1:100), rabbit anti-claudin-5 antibody (1:100), rabbit anti-p120-Catenin (1:200), rabbit anti-β-Catenin (1:100), anti-CD31(1:100), anti-BrdU (1:100) and anti-Laminin (1:100) at 4 °C. This was followed by incubation with Alexa Fluor 488 Affinipure goat anti-rabbit IgG (H + L) (1:200, Yeasen, China) for 50 min at room temperature. Phosphate-buffered saline (PBS) was used in place of the primary antibody as a negative control. We use an in situ cell death detection kit to detect apoptotic cells. The nuclei were coloured by staining with Hoechst or DAPI. The fluorescence signal was observed by laser confocal microscopy. Five fields on each of three slides per animal were randomly selected for visualization and analysis performed using ImageJ software (National Institutes of Health, Bethesda, MD, USA).

### Transmission electron microscopy (TEM)

A total of 18 mice were subjected to TEM. Tissue was quickly removed, cut into 1 mm^3^ pieces on ice and soaked in 2.5% glutaraldehyde. The tissue was then fixed with a 1% oxidizing fixative for 1 h, stained with 1% uranyl acetate for 2 h, and embedded after dehydration in a gradient acetone solution. After semi-thin sectioning and toluidine blue staining, ultrathin sections were cut and observed using a Hitachi transmission electron microscope.

Images were taken under the same conditions, including brightness and contrast, to better compare TJs among different groups. According to standard protocols [30, 31], the width and length of the TJs were blindly measured and averaged by two examiners using ImageJ software.

### Statistical analyses

All experimental data are expressed as the mean ± standard deviation. The Kolmogorov–Smirnov (K–S) test was used as a normality test, with *p* > 0.05 indicating a normal distribution, Levene’s test was used as a test of homogeneity of variance, with *p* > 0.05 used to indicate homogeneous variance, and vice versa. A t-test was used to compare two groups. One-way ANOVA and Dunnett’s test were used to evaluate the data when more than two groups were compared. Statistical analyses were performed with SPSS 16 statistical software, and *p* < 0.05 was used to indicate statistical significance.

## Results

### TT reduced permeability of the BSCB after SCI

**Fig. 1:**
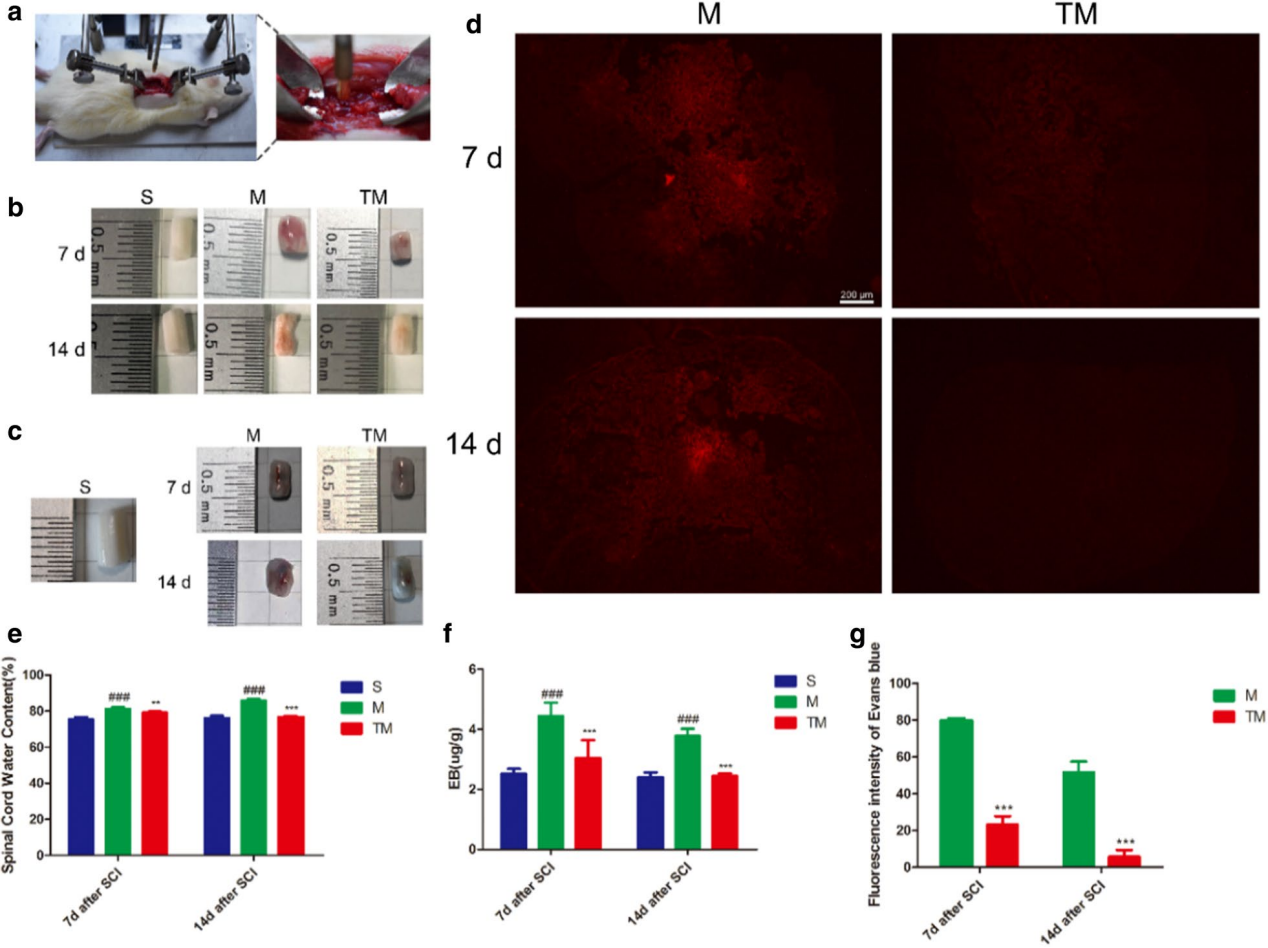
TT reduced the influx of water and EB dye after SCI. **a** An SCI model was generated with an NYU Impactor (10 g × 20 cm). **b** Representative views of an excised T10 cord. **c** Representative views of EB dye permeabilized into excised spinal cord tissue after SCI. **d** Representative fluorescent images showing EB dye extravasation(scale bar = 200 µm). **e** Quantification of water content in the S (sham-operated), M (SCI) and TM (SCI + TT) groups; *columns* represent the mean ± SD (n = 5). **f** Quantification of EB dye in the tissue (µg/g) (n = 5). **g** Quantification of the fluorescence intensity (n = 5). ^#^*p* < 0.05 for the M group versus the S group, *p < 0.05 for the TM group versus the M group. (^#^*p*, *p < 0.05; ^##^*p*, **p < 0.01; ^###^*p*, ***p < 0.001)

We used an impactor to generate a model of SCI (Fig. 1a). Subsequently, EB staining and analysis of the spinal cord water content were performed to determine the degree of tissue damage. The results showed that the water content increased significantly after SCI and that TT could reduce the oedema caused by SCI at both 7 and 14 d after SCI (Fig. 1b, e) (M vs. S: *p*_*7*_ < 0.001, *p*_*14*_ < 0.001; TM vs. M: *p*_*7*_ < 0.01, *p*_*14*_ < 0.001). The amount of EB dye in the tissue increased significantly after SCI compared with that in group S, suggesting BSCB leakage (M vs. S: *p*_*7*_ < 0.001, *p*_*14*_ < 0.001). After TT, infiltration of EB dye was significantly reduced (Fig. 1c, f) (TM vs. M: *p*_*7*_ < 0.001, *p*_*14*_ < 0.001). The fluorescence intensity of the EB dye after SCI was much higher than that in the S group. However, the fluorescence level of the TM group was significantly lower than that of the M group (Fig. 1d, g) (TM vs. M: *p*_*7*_ < 0.001, *p*_*14*_ < 0.001). All these data indicate that TT could reduce BSCB disruption.

### TT decreased tissue structural damage and improved functional recovery after SCI

**Fig. 2:**
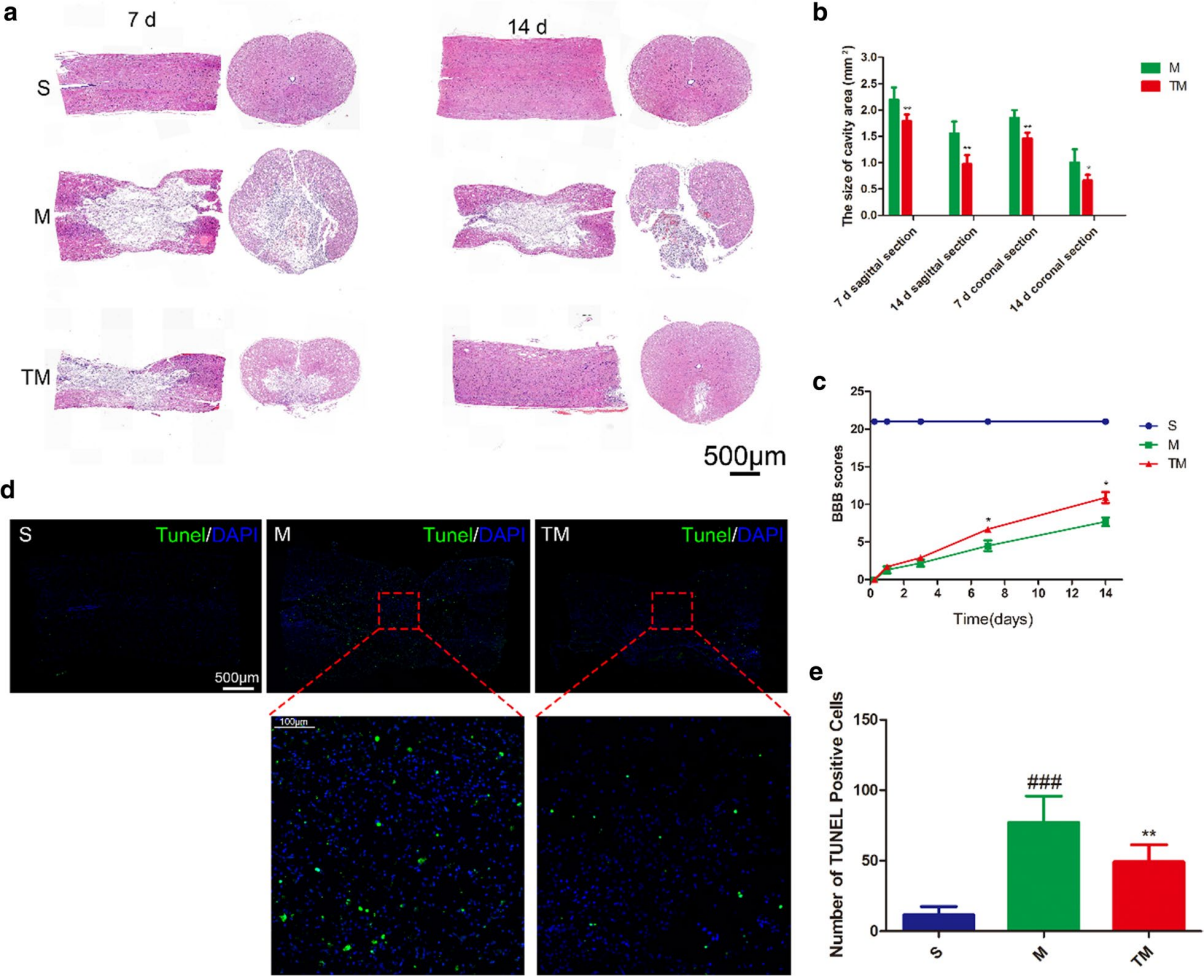
TT decreased tissue structural damage and improved functional recovery after SCI. **a** HE-stained sections at 7 and 14 d after SCI. Scale bars = 500 µm. **b** Quantification of the cavity area; *columns* represent the mean ± SD (n = 5). **c** BBB scores in the S, M, and TM groups. **d** TUNEL staining in the epicentre at 7 d after SCI. Scale bars represent 500 µm and 100 µm. **e** Quantitative estimation of apoptotic cells. *Columns* represent the mean ± SD (n = 5) (^#^*p*, *p < 0.05; ^##^*p*, **p < 0.01; ^###^*p*, ***p < 0.001)

At 7 and 14 d after injury, histological differences in the T9-T11 spinal cord were observed by HE staining (Fig. 2a). The arrangement of tissues in group S was normal. In group M the grey matter and white matter were destroyed to varying degrees, accompanied by the death of multiple neurons (Fig. 2e). In group TM, the tissue structure was significantly better than that in group M. Quantitative analysis of the cavity area showed the same results (Fig. 2b) (TM vs. M: coronal section, *p*_*7*_ < 0.01, *p*_*14*_ < 0.05; sagittal section, *p*_*7*_ < 0.01, *p*_*14*_ < 0.01). The functional recovery of the S, M and TM groups was evaluated by BBB motor functional scoring at 6 h, 1 d, 3 d, 7 d, and 14 d after SCI (Fig. 2c). The BBB scores of the TM group were significantly higher than those of the M group at 7 and 14 d after SCI (TM vs. M: *P*_*7*_ < 0.05, *P*_*14*_ < 0.05). We further tested the relationship between apoptosis and TT at 7 d after SCI (Fig. 2d). The number of apoptotic cells (TUNEL staining) in the epicentre increased significantly after SCI. Compared with the M group, the number of apoptotic cells in the TM group was significantly decreased (M vs. S: *P* < 0.001; TM vs. M: *P* < 0.01). These results show that TT could significantly improve functional recovery and preserve tissue.

### TT prevented the loss of TJ and AJ proteins

**Fig. 3:**
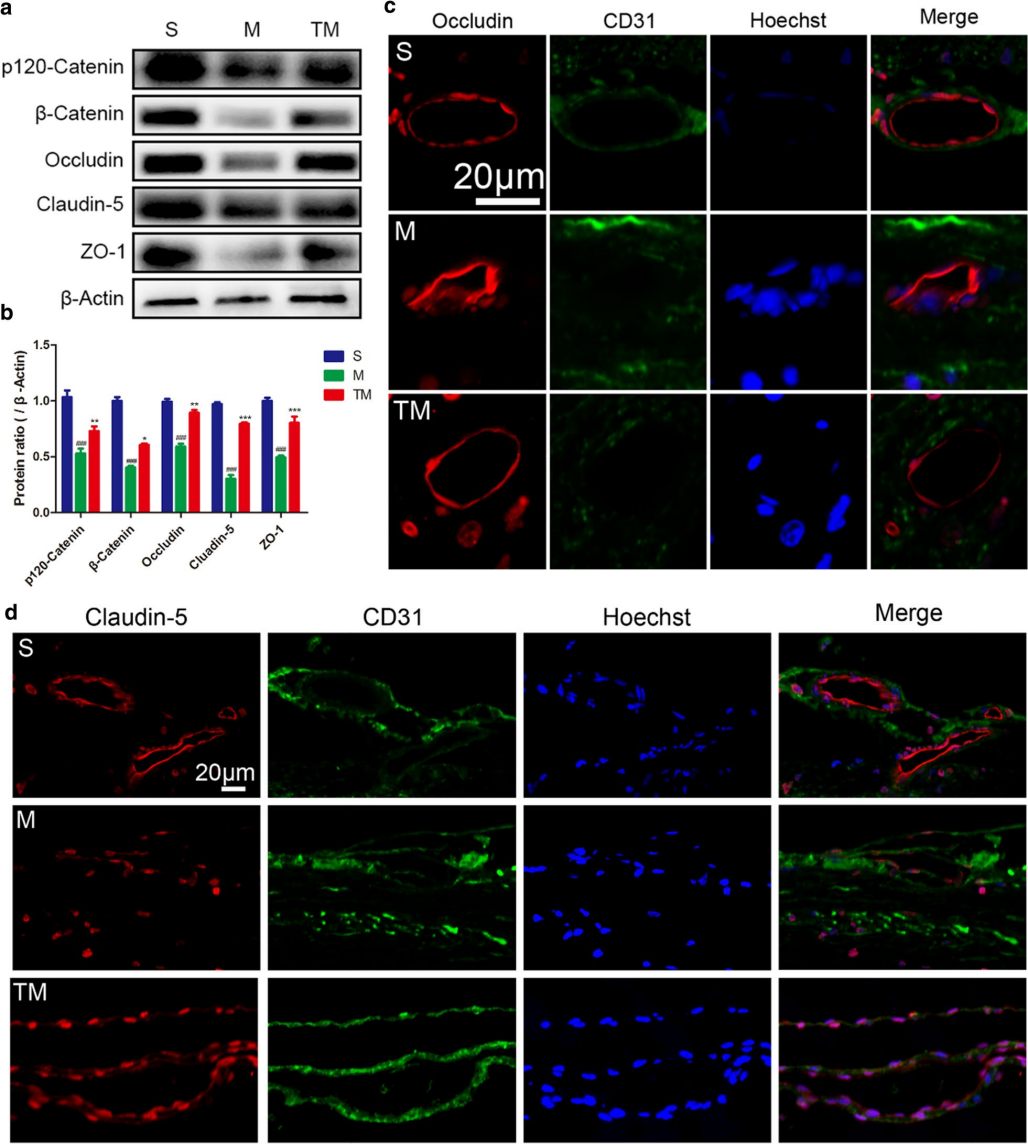
TT prevented the loss of TJ and AJ proteins. Representative **a** Western blots and **b** quantification of TJ and AJ proteins in each group; *columns* represent the mean ± SD (n = 5). Double staining for **c** Occludin/CD31/Hoechst and **d** Claudin-5/CD31/Hoechst. Red: Occludin/Claudin-5; green: CD31; blue: Hoechst. Scale bar, 20 µm (^#^*p*, *p < 0.05; ^##^*p*, **p < 0.01; ^###^*p*, ***p < 0.001)

**Fig. 4:**
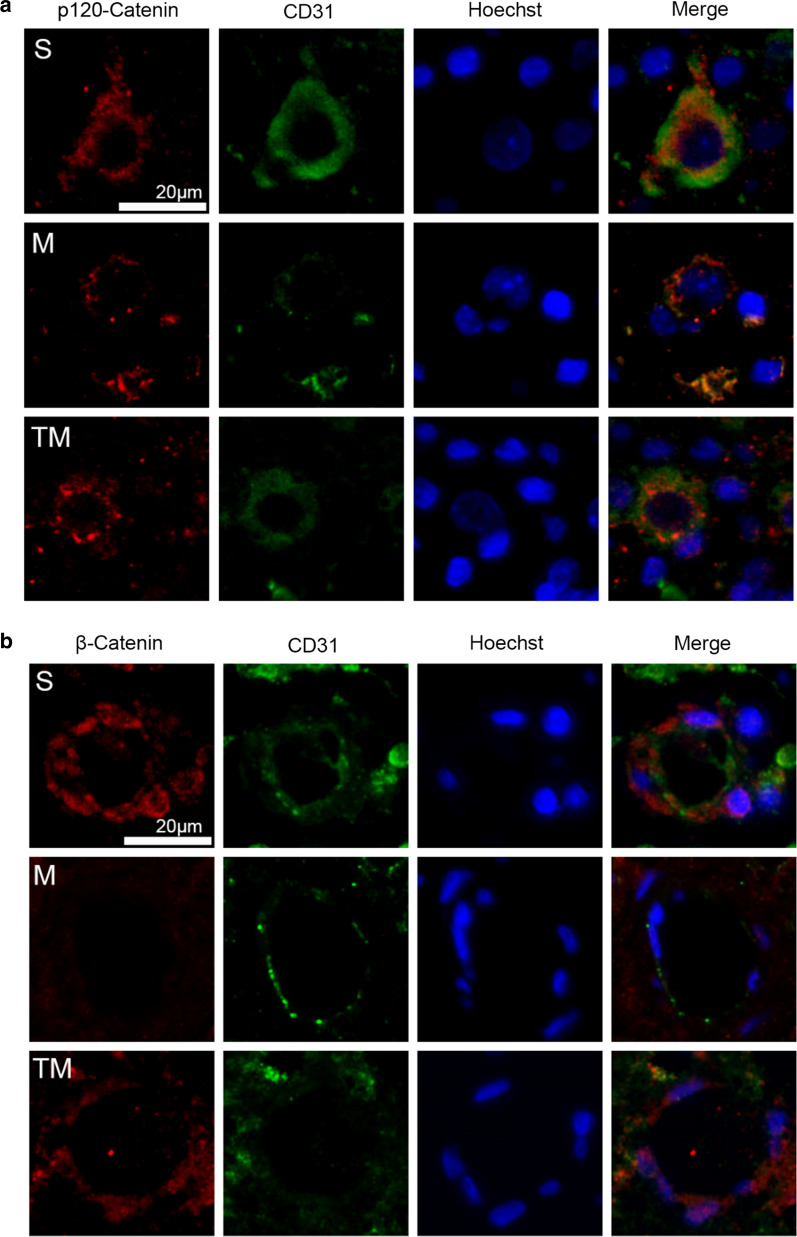
Double staining for **a** p120-Catenin/CD31/Hoechst and **b** β-Catenin/CD31/Hoechst. red: p120-Catenin/β-Catenin; green: CD31; blue: Hoechst. Scale bar, 20 µm

To further determine the effects of TT on interendothelial junctions, AJ proteins (β-Catenin and p120-Catenin), TJ proteins (Claudin-5 and Occludin) and ZO-1 expression was estimated by Western blotting. According to the results (Fig. 3a), TJ and AJ protein expression was decreased to some extent after SCI compared to the levels of the control group (M vs. S: β-Catenin, p120-Catenin, Claudin-5, Occludin ZO-1, *p* < 0.001). However, compared to the rats in M group, rats treated with TT showed higher TJ and AJ protein expression at 7 days (Fig. 3b) (TM vs. M: β-Catenin,* p* < 0.05; p120-Catenin, *p* < 0.01; Claudin-5, *p* < 0.001; Occludin, *p* < 0.01; ZO-1, *p* < 0.001). Staining for Occludin/CD31/Hoechst (Fig. 3c), Claudin-5/CD31/Hoechst (Fig. 3d), p120-Catenin/CD31/Hoechst (Fig. 4a) and β-Catenin/CD31/Hoechst (Fig. 4b) was used to show the distribution of BSCB proteins after SCI and indicated that TT reduced the degradation of Claudin-5/Occludin/p120-Catenin/β-Catenin around the epicentre. These results suggest that TT could prevent the loss of TJ and AJ proteins after SCI.

### TT promoted angiogenesis after SCI

**Fig. 5:**
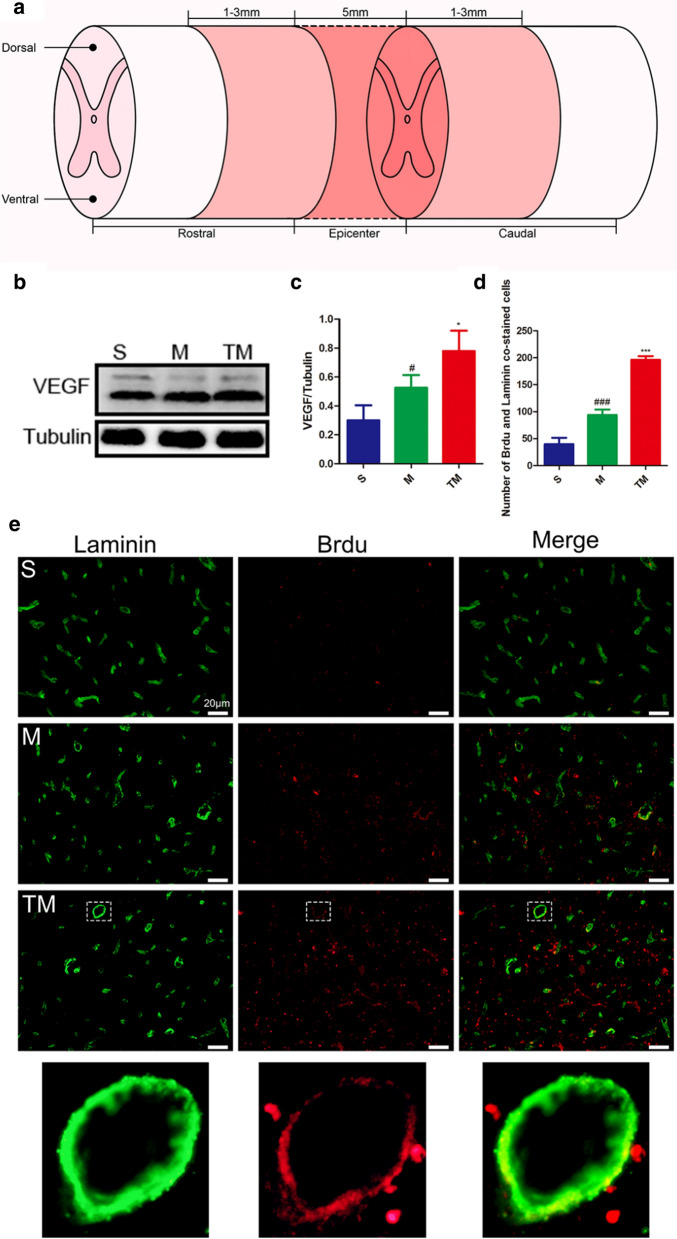
TT promoted angiogenesis after SCI. **a** Schematic diagram of the regions sampled after SCI. **b** Representative Western blots and **c** quantification of VEGF/Tubulin; *columns* represent the mean ± SD (n = 5). **d** Quantification of the number of cells co-stained for BrdU and Laminin; *columns* represent the mean ± SD (n = 5). **e** Double staining of sections from the spinal cord in each group of rats for Laminin (green)/BrdU (red). Scale bars represent 20 μm (^#^*p*, *p < 0.05; ^##^*p*, **p < 0.01; ^###^*p*, ***p < 0.001)

We investigated VEGF protein expression in the epicentre at 7 d after SCI (Fig. 5a). Compared with VEGF expression in group S, VEGF expression increased significantly after SCI (M vs. S: *p* < 0.05). Furthermore, TT significantly upregulated VEGF expression compared to that in rats that received SCI alone (Fig. 5b, c) (TM vs. M: *p* < 0.05). Then, blood vessels co-labeled with 5-bromo-2-deoxyuridine (BrdU) and laminin in the ischaemic penumbra (the light shaded part in Fig. 5a) were quantified. As shown in Fig. 5d, e, angiogenesis in the M group was significantly higher than that in the S group at 7 d after injury (M vs. S: *p* < 0.001). Additionally, the neovascularization density of the TM group was increased compared with that in the M group, suggesting that TT could effectively promote angiogenesis (TM vs. M: *p* < 0.001).

### T inhibited MMP-2/9 expression after SCI

**Fig. 6:**
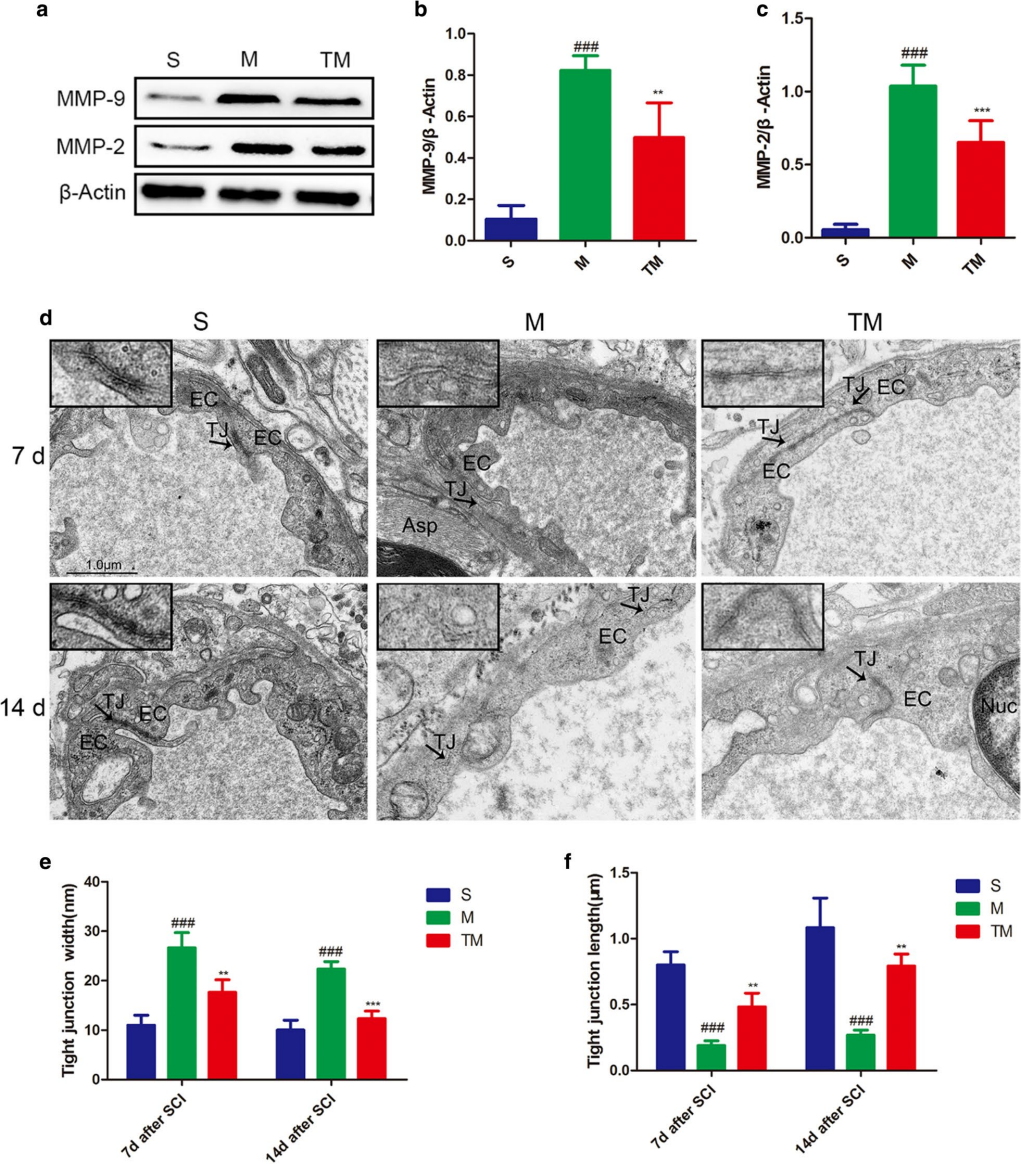
TT inhibited increased MMP-2 and MMP-9 expression after SCI. **a**–**c** Representative Western blots and quantification of MMP-2/9 7 d after SCI in each group; *columns* represent the mean ± SD (n = 5). **d** TEM shows EC-EC junctions in the S, M, and TM. Arrows indicate TJ electron-dense bands and open TJs. Scale bars = 1.0 µm. **e** Quantification of the TJ gap width among the S, M, and TM groups; *columns* represent the mean ± SD. **f** Quantification of the TJ length among the S, M, and TM groups; *columns* represent the mean ± SD. (^#^*p*, *p < 0.05; ^##^*p*, **p < 0.01; ^###^*p*, ***p < 0.001)

MMP-2/9 protein expression levels were detected by Western blotting, which showed that TT could significantly inhibit the upregulation of MMP-2/9 after SCI (Fig. 6a–c) (TM vs. M: MMP-2, *p* < 0.001; MMP-9, *p* < 0.01). Moreover, to detect defects in vascular ECs, we analysed them by TEM. In group S, spinal cord vascular ECs showed tight connections with a rivet-like structure, and a series of electron-dense bands between the plasma membranes, forming a relatively closed vascular lumen, were observed. The intercellular TJs were partially opened after SCI (M group), and compared with that in group S under the same conditions, the structure of the junctions was more disordered; for example, the TJ length was shorter and the gap was wider (M vs. S: TJ width, *p*_*7*_ < 0.001, *p*_*14*_ < 0.001; TJ length, *p*_*7*_ < 0.001, *p*_*14*_ < 0.001). In contrast, rats in the TM group had more electron-dense bands, and the ultrastructural changes in these rats were between those observed in the S and M groups (Fig. 6d–f) (TM vs. M: TJ width, *p*_*7*_ < 0.01, *P*_*14*_ < 0.001; TJ length, *p*_*7*_ < 0.01, *P*_*14*_ < 0.01).

## Discussion

Our data in this study indicate that TT can protect the integrity of the BSCB and prevent further spinal cord oedema after impact injury. After TT, the histological structure and motor function of the spinal cord were significantly improved. We showed that TT could promote angiogenesis, reduce apoptosis and inhibit MMP-2/9 expression. Therefore, the vascular protection of the BSCB by TT may occur through the following mechanisms: TT (1) protects the residual BSCB structure from further damage, (2) promotes vascular regeneration, and (3) inhibits MMP-2/9 expression to mitigate BSCB damage.

Emerging evidence indicates that exercise therapy can promote recovery after SCI [32–35]. It is well known that normal exercise training cannot be carried out by rats after SCI. Thus, we introduced water TT, which simulates clinical exercise treatment and can help rats successfully train on a treadmill in the early stage of SCI. TT for human patients is mainly used in rehabilitation for motor system diseases [36, 37]. TT can reduce the resistance of forward movement, allowing patients to walk or run with a normal gait [38]. Additionally, the depth and speed of TT can be adjusted, which facilitates the control of exercise intensity [38].

For the first time, mechanisms to mitigate damage after SCI were studied through our experiments in rats. Trauma, infection, tumour growth or obstruction of the blood supply can cause oedema of the central nervous system (CNS). Cytotoxicity and vasogenic edema are interdependent factors involved in the development of CNS oedema. Prolongation of cytotoxic oedema leads to vasogenic oedema, and vice versa [39, 40]. Cytotoxic oedema, which refers to the accumulation of water in intact cells, occurs when an anoxic state leads to the loss of energy-dependent solute homeostasis [41]. Vasogenic oedema refers to the accumulation of fluid in the extracellular space around the damaged BBB/BSCB [42]. The integrity of the BSCB is essential for the spinal cord to maintain its normal function [43]. Destruction of the BSCB after SCI, leads to increased permeability, causing secondary damage [18, 44]. In our study, through the horizontal comparison of data from each group collected at the same time point, we found that the degree of oedema in the SCI group was increased significantly higher than that in the sham-operated group; this was accompanied by lower BBB motor scores, severe tissue structure damage and a large number of necrotic cells. However, these conditions were significantly improved after TT, which may be related to the protective effect of TT on the BSCB. Longitudinal comparison of the BBB scores 7 and 14 d after SCI showed some recovery with time (Fig. 2c). The partial recovery of these functions may be related to both the preservation of BSCB function and formation of new blood vessels.

The BSCB protects the spinal cord by restricting the entry of plasma components and blood cells [9]. Following SCI, destruction of the BSCB leads to increased microvascular permeability, inflammatory reactions, tissue oedema, and neurotoxic products [45]. TJs, AJs, and gap junctions connect the ECs lining microvessels in the spinal cord [3, 46]. The dense network of TJs and AJs is destroyed after SCI, resulting in the decreased expression of TJ and AJ proteins. Our results show thatp120-Catenin, β-Catenin, ZO-1, Occludin, and Claudin-5 expression was greatly reduced after SCI. However, their expression was significantly improved in the TT-treated spinal cord (Figs. 3, 4). This shows that the residual BSCB structure had been protected by TT.

To determine the protective mechanism of TT, we performed more in-depth research. VEGF stimulates EC proliferation and survival vascular structure formation, nitric oxide-dependent vasodilatation and vascular leakage [17]. It has been reported that VEGF peaks at 3 d, and a large amount of VEGF remained at 7 d after SCI [47]. In addition, ischaemia and injury can induce angiogenesis, which provide oxygen and nutrition to the ischaemic or diseased site, thus improving tissue repair and remodeling [48–50]. We used laminin as a vascular marker and BrdU as a proliferation marker [51]. The results showed that TT could promote angiogenesis after SCI, as shown by the detection of VEGF protein expression. Quantitative analysis of neovascularization 7 d after SCI with co-labelling also showed that the number of BrdU^+^/Laminin^+^ cells increased significantly after TT treatment in rats with SCI, helping to maintain the stability of the BSCB (Fig. 5).

The activation of MMP-2/9 after SCI plays an essential role in the destruction of the BBB/BSCB [9, 10], and MMP-2/9 expression can aggravate damage [52]. In this study, 7 d after SCI, MMP-2/9 expression was upregulated (Fig. 6a–c), showing their potential to further aggravate and damage the BSCB. We were surprised to find that MMP-2/9 expression was significantly decreased after TT. Although we have not proven how TT downregulates MMP-2/9 expression, we believe that TT can effectively prevent the destruction of the BSCB, partially through the inhibition of MMP-2/9 expression after SCI.

As far as we know, this is the first time that TT has been applied in the treatment of SCI in rats. We found that TT could enhance the expression of TJ and AJ proteins after SCI. We also found that TT could promote angiogenesis and inhibit MMP-2/9 expression, which may be an important mechanism by which TT maintains the stability of the BSCB. These experimental results provide a better understanding of the possible mechanisms of TT in the treatment of SCI and a reliable basis for the application of TT in the future.

We acknowledge the limitations of our experiments. In this study, we only used male rats, ignoring any possible differences caused by sex. Due to limitations in the experimental conditions, we could not observe changes at the functional level through electrophysiological techniques to understand the role of TT. The training period was relatively short and the specific mechanism by which TT inhibits MMP-2/9 expression remains to be further studied.

## Conclusion

**Fig. 7:**
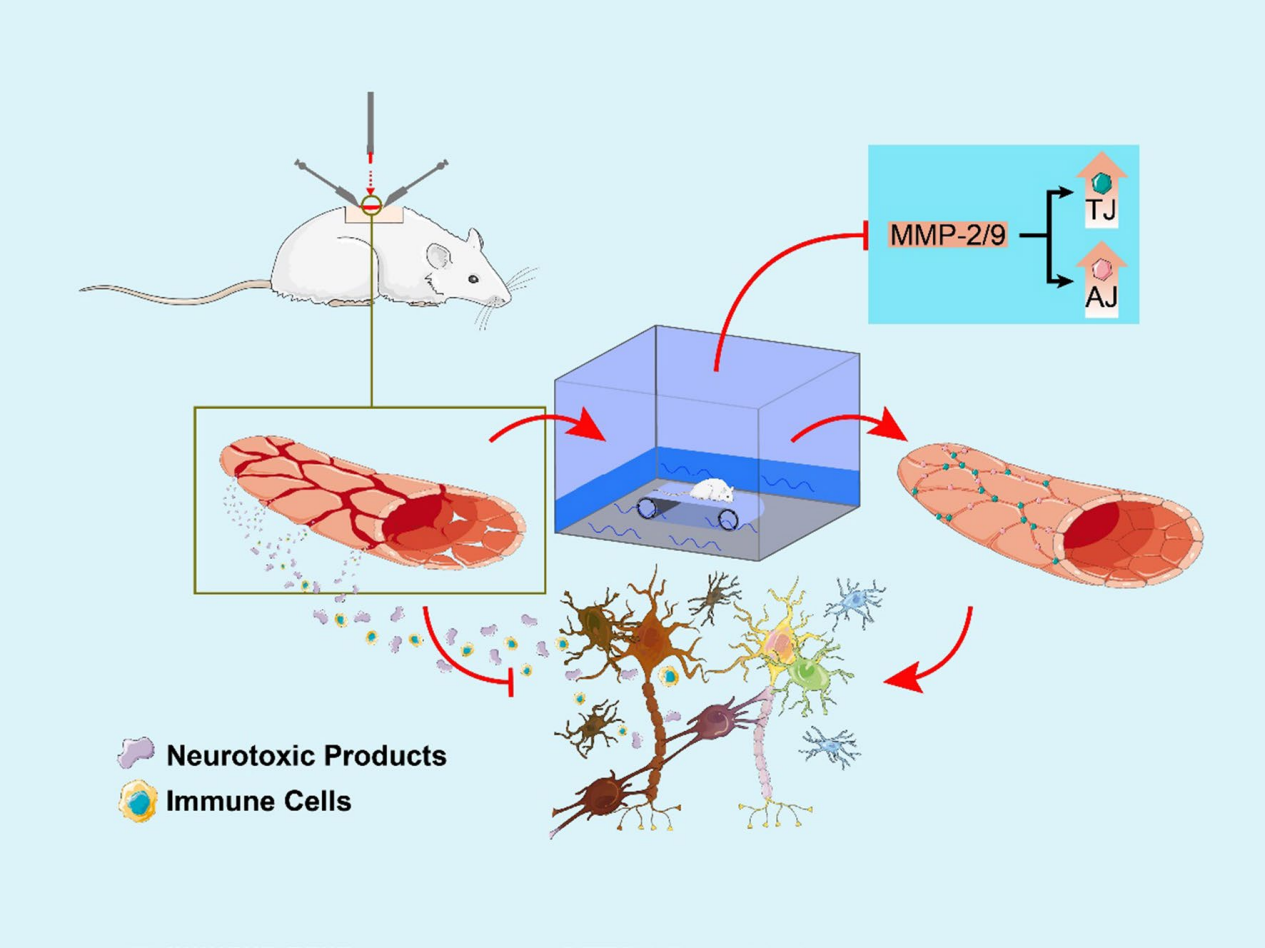
Proposed mechanism by which water TT protects the BSCB after SCI. Barrier integrity is compromised by the disruption of interendothelial TJs and AJs, as well as overall mechanical damage to vessels after SC. This results in the infiltration of immune cells and neurotoxic products, causing nerve cell death. Water treadmill training prevents disruption of the BSCB by protecting the residual BSCB structure, promoting vascular regeneration and inhibiting MMP-2/9 expression following SCI

TT participates in protecting the BSCB after SCI by protecting the residual BSCB structure from further damage. Furthermore, TT also promotes vascular regeneration and inhibits MMP-2/9 expression to mitigate BSCB damage (Fig. 7). However, TT-induced BSCB protection after SCI is a complex process that involves many key factors and remains to be fully elucidated. Due to the aforementioned limitations of our study, our next research direction will be an in-depth study of the upstream molecular mechanisms of MMP-2/9 and VEGF, using techniques such as in vivo electrophysiology to study the spinal cord at the functional level.

## Supplementary information

**Additional file 1.** The movement of the rats on the water treadmill.

## Data Availability

The datasets supporting the conclusions of this article are available from the corresponding author on reasonable request.

## Abbreviations

SCI: Spinal cord injury
TT: Treadmill training
BSCB: Blood-spinal cord barrier
TJ: Tight junction
AJ: Adherens junction
MMP: Matrix metalloproteinase
BBB: Basso-Beattie-Bresnahan
HE: Haematoxylin–eosin
EB: Evans blue
WB: Western blotting
TEM: Transmission electron microscopy
VEGF: Vascular endothelial growth factor
PBS: Phosphate-buffered saline
BrdU: 5-Bromo-2-deoxyuridine
EC: Endothelial cell
CD31: Platelet endothelial cell adhesion molecule-1
CNS: Central nervous system
